# Probiotic Beverage with Soy Isoflavone Consumption for Breast Cancer Prevention: A Case-control Study

**DOI:** 10.2174/15734013113099990001

**Published:** 2013-08

**Authors:** Masakazu Toi, Saya Hirota, Ai Tomotaki, Nobuaki Sato, Yasuo Hozumi, Keisei Anan, Takeshi Nagashima, Yutaka Tokuda, Norikazu Masuda, Shozo Ohsumi, Shinji Ohno, Masato Takahashi, Hironori Hayashi, Seiichiro Yamamoto, Yasuo Ohashi

**Affiliations:** Department of Biostatistics, School of Public Health, The University of Tokyo, 7-3-1 Hongou, Bunkyo-ku, Tokyo, 113-0033 Japan

**Keywords:** Breast cancer, *Lactobacillus casei* Shirota, probiotic beverage, soy isoflavones.

## Abstract

The purpose of this study is to evaluate how beverages containing *Lactobacillus casei* Shirota (BLS) and soy isoflavone consumption since adolescence affected the incidence of breast cancer. In a population-based case-control study, three hundred and six cases with breast cancer and 662 controls aged 40 to 55 were matched for age and residential area and included in the analyses. Diet, lifestyle and other breast cancer risk factors were investigated using the self-administered questionnaire and interview. Odds ratios (ORs) of BLS and soy isoflavone consumption for breast cancer incidence were independently and jointly estimated using a conditional logistic regression. The ORs of BLS consumption (≥ four times a week against < four times a week) was 0.65 and statistically significant (p = 0.048). The analysis of association between soy consumption and breast cancer incidence showed the more the isoflavone consumption is, the lower the odds of breast cancer becomes. Adjusted ORs for breast cancer in the second, the third and the fourth quartiles of soy consumption against the first quartile were 0.76, 0.53 and 0.48, respectively (trend test, p = 0.0002). The BLS-isoflavone interaction was not statistically significant; however, a biological interaction was suggested. Regular consumption of BLS and isoflavones since adolescence was inversely associated with the incidence of breast cancer in Japanese women.

## 1 INTRODUCTION

The breast cancer incidence in Japan had been lower compared to the Western countries; however, rapid increase of the incidence was observed in the past 10 to 15 years as in other Asian countries [1]. The number of new breast cancer cases in Japanese women surpassed that of stomach cancer to become the most frequent of all cancers in 1994. An estimated 45700 women were newly diagnosed with breast cancer in 2003 (an estimated 36500 with stomach cancer) [2]. One of the likely causes of the rapid increase in Japan is the increased estrogen exposure [3], one of the important breast cancer risk factors, which is due to delayed first delivery and decreased number of child births. However, only about 40% of the increase can be explained by age of menarche, age of menopause, age of having the first child, and number of births. Changes in the traditional Japanese lifestyle and increase of obesity are possible contributing factors [4].

Beverages containing *Lactobacillus casei *Shirota (BLS, Yakult^®^, Yakult Honsha, Co. Ltd., Tokyo, Japan) have been sold in Japan since 1935. According to the manufacturer’s data, 84% of BLS were sold through a unique personal home and office delivery system in 1985, and the product took up an estimated 50% or more of the Japanese fermented dairy product market in 1970s and 1980s.

A Japanese case-control study showed regular consumption of BLS was inversely associated with occurrence of bladder cancer [5]. *Lactobacillus casei *Shirota was shown to prevent recurrence of colon polyp in a randomized trial [6] and to prevent recurrence of superficial bladder cancer in two randomized trials [7, 8]. The cancer preventive effect of BLS may be explained by potentiation of natural killer (NK) cell activation by *Lactobacillus casei *Shirota and NK cell-mediated antitumor activity [9].

The association between soy consumption and occurrence of breast cancer has been evaluated in a number of epidemiological studies. Studies in Japan showed that soy consumption was inversely associated with occurrence of breast cancer in a cohort study by Yamamoto and colleagues [10], and Hirose [11] reported the same association in a hospital-based case-control study. A meta-analysis conducted by Wu and colleagues [12] also showed an inverse association when the analysis included only studies conducted in Asian countries where soy consumption is high.

The mechanism of soy to prevent breast cancer may be ascribed to estrogenic and anti-estrogenic actions of isoflavones such as genistein and daidzein contained in soy. In a nested case-control study conducted along with the Japanese cohort study [13], an inverse association between serum isoflavone level and occurrence of breast cancer was reported. Intestinal flora is known to affect the isoflavone metabolism, especially the metabolism of an isoflavone phytoestrogen daidzein [14].

On the other hand, consumption of BLS was shown to affect the intestinal flora in a randomized double-blind study [15], suggesting BLS consumption may modify the preventive effect of soy isoflavones against breast cancer.

Based on these findings, we evaluated the role of probiotic beverages in breast cancer prevention in Japanese women with implication of soy isoflavone consumption in a population-based case-control study. The study is to explore the cause of the recent rapid increase of breast cancer in Japan and the possibility of breast cancer prevention by lifestyle modification.

## 2 MATERIALS AND METHODS 

### 2.1 Study Setting and Participants

This population-based case-control study took place in 14 areas in Japan. The catchment areas were selected so that both rural and urban areas were covered. Cases were defined as Japanese women aged 40 to 55 at the time of consent, who had undergone operation for the International Union against Cancer Tumor Node Metastasis (UICC-TNM) Stage 0 or I unilateral or bilateral primary breast cancer at one of 14 study centers within one year prior to participation. Women who had breast cancer of UICC-TNM Stage II or higher and those with other types of cancer were excluded.

Controls were Japanese women living in the catchment areas aged between 40 and 55 without past or present breast cancer. Women aged 40 to 55 were first picked out from the catchment areas based on the Basic Resident Register, and potential controls were randomly selected. Once a case was included in the study, controls matched to the case for age (the range of target age was divided into two-year brackets) and residential area were randomly selected from the pool of candidate controls and invited to participate. Invitation letters were sent until two controls were selected for a case.

The study was approved by the institutional review board of all study centers. Cases and controls were recruited between October 9, 2007 and March 31, 2009. Informed consent was obtained from all cases and controls enrolled in the study.

### 2.2 Procedures

A self-administered questionnaire survey and interviews were conducted. To avoid recall biases, participants were asked about their past BLS and soy consumption in interviews. Frequency and duration of BLS consumption and frequency and amount of soy (six items including miso-soup and tofu) consumption during three predetermined time periods; around the age 10 to 12, around the age 20, and 10 to 15 years prior to the study; were asked in the questionnaire. We conducted a self-administered questionnaire survey to ask about their diet (including alcohol consumption), exercise, medical history, and family history during the year before their breast cancer diagnosis to cases and during the year before the survey to controls. We used a self-administered food frequency questionnaire created based on the food frequency questionnaire developed by the Japan National Cancer Center (JNCC) [16], of which validity has been confirmed in the previous study showing the effect of soy consumption in the prevention of breast cancer [10].

Cases and matched controls were interviewed by the same trained interviewer who was blinded to the case/control status. A set of self-administered questionnaire forms was handed to each participant at the time of consent. Completed questionnaire forms were kept by participants until the interview and checked by interviewers for omission.

### 2.3 Exposure Assessment

BLS and isoflavone consumption in the three predetermined time periods was averaged for the purpose of analysis. BLS consumption was classified into regular consumption (≥ 4 times a week) and no consumption (< 4 times a week). Isoflavone consumption was calculated based on the frequency and the amount of the six food items and classified into quartiles according to the daily consumption in the control group: > 43.75 mg/day (the fourth quartile), 28.81 to 43.75 mg/day (the third quartile), 18.76 to 28.81 mg/day (the second quartile), and < 18.76 mg/day (the first quartile).

### 2.4 Statistical Analysis

Based on a hypothesized OR of 0.55 and a 15% prevalence of exposure to BLS in the control group, the target sample size was calculated to be 355 for cases and 710 for controls to achieve a 80% power with a 5% two-sided type I error. We calculated ORs and 95% confidence intervals (CIs) using a conditional logistic regression model. P-values for potential dose-response of soy isoflavone consumption on breast cancer were calculated based on the same regression model using linear scores. We also performed a subgroup analysis of association between BLS consumption and breast cancer occurrence according to menopausal status.

Cases and controls were matched for age and area of residence for one analysis and for area of residence alone for a separate analysis. Robustness of the results was also examined in a sensitivity analysis using an unconditional logistic regression model, which adjusted the matching variables as covariates. All p-values were two-sided.

Education, physical activity level, history of benign mammary tumor, family history of breast cancer, past/current use of female sex hormones before menopause, age at menarche, number of childbirth, breastfeeding experience, birth weight, BMI at the age 20, smoking status, and current energy intake were taken into account as potential confounders.

We used the SAS LOGISTIC procedure (version 9.1; SAS Institute, Cary, NC) for all analyses. 

### 2.5 Role of the Funding Source

The sponsor organized the study group under one of the projects of Comprehensive Support Project for Oncology Research (CSPOR) [17, 18]. The independent ethics committee and the epidemiology committee of CSPOR approved the study protocol. The funding source of the study, Yakult Honsha Co. Ltd., was not involved in the study design, data collection, data analysis, data interpretation or writing of the study reports. The corresponding author had full access to all the study data and had final responsibility for the decision to submit the study report for publication.

## 3 RESULTS

**Fig. (1). Study profile.**
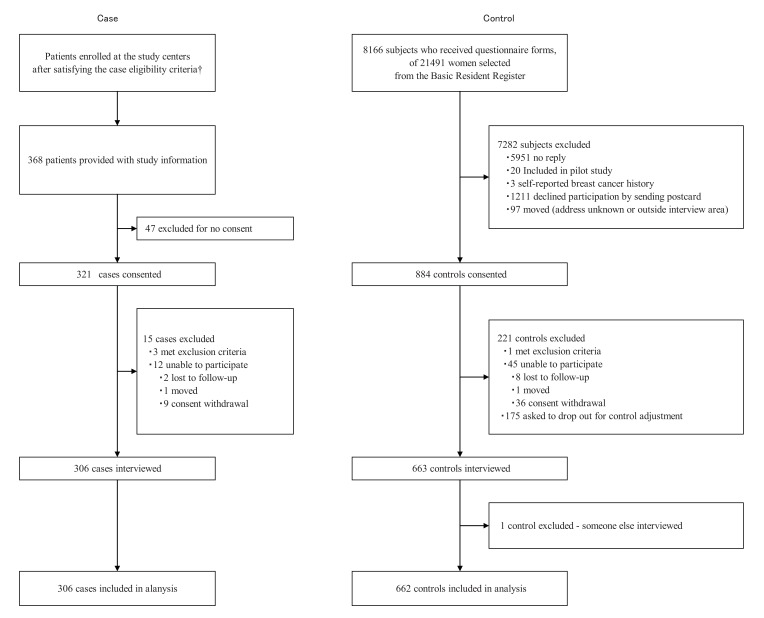
†: Data for the number patients screened are not available.

(Fig. **1**) presents a flow chart of the case and control recruitment process. One control was excluded because we later found out her mother had filled out the questionnaire forms and had been interviewed. Baseline characteristics of 968 participants, including two cases and 32 controls not matched for age due to some adjustment in the sampling procedure, are shown in (Table **1**). Statuses of estrogen receptor, progesterone receptor and human epidermal growth factor receptor in cases are shown in (Table **2**).

Crude ORs (matched for age and area of residence) and adjusted ORs (matched for area of residence and adjusted for the age and other confounding factors) for breast cancer in women who had consumed BLS ≥ four times per week against those who had consumed < four times per week were 0.66 (95% CI, 0.43–1.02; p = 0.061) and 0.65 (95% CI, 0.42–1.00; p = 0.048), respectively. (Table **3**) Crude ORs for isoflavone consumption in the second, the third and the fourth quartiles against the first quartile were 0.74 (95% CI, 0.49–1.10), 0.58 (0.38–0.88) and 0.52 (0.34–0.79), respectively. (Trend test, p = 0.0012) Adjusted ORs were 0.76 (0.52–1.13), 0.53 (0.35–0.81) and 0.48 (0.31–0.73), respectively (trend test, p = 0.0002; Table **3**).

We present the results of adjusted analysis based on a model in which participants were matched for residential area and age and categorized into the age brackets of 40s and 50s. Categorical age was used as an explanatory variable. The number of participants included in the analysis was maximized with this model. The results were mostly comparable to those of a logistic regression analysis in which the matching factors were adjusted as covariates.

Significant breast cancer risk factors used for adjustment included above high school-level education (adjusted ORs, 0.61 for the BLS analysis and 0.63 for the isoflavone analysis), benign tumor (adjusted ORs, 3.0 and 3.2), and family history of breast cancer (adjusted ORs, 2.1 and 2.2).

**Fig. (2). Interaction between probiotic beverage and soy isoflavone consumption.**
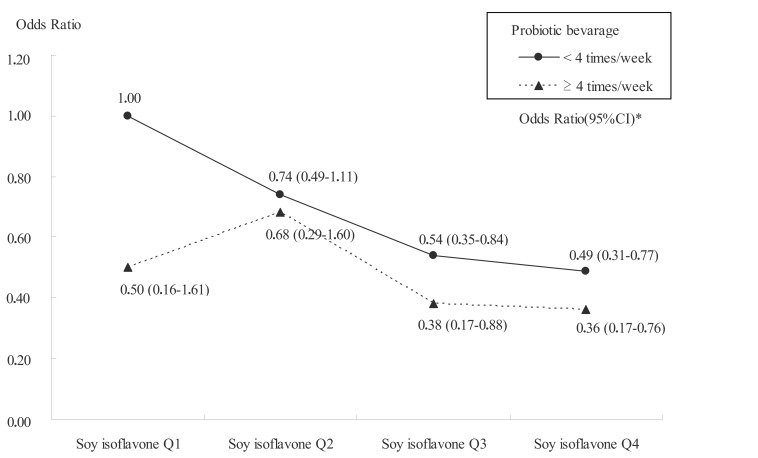
* Calculated using conditional logistic regression. 306 cases and 661 controls were matched for residential area and adjusted for age (40s and 50s), educational background, physical activity level, benign mammary tumor, family breast cancer history, menarche, number of births, breastfeeding, use of female hormone, birth weight, Body Mass Index around the age of 20, smoking and energy intake. One control was excluded due to lack of adjustment factor.

According to an analysis using a multiplicative factor, the interaction between BLS and isoflavones was not statistically significant (p = 0.87) but there was a trend of weaker dose-response relationship between isoflavone consumption and breast cancer as observed from the flat curve in women who consumed more BLS (Fig. **2**).

A subgroup analysis according to menopausal status (matched for area of residence and adjusted for age and other confounding factors) showed an adjusted OR in premenopausal women was 0.78 (95% CI, 0.46–1.32; p = 0.35) and that in postmenopausal women was 0.43 (0.19–0.99, p＝0.046) (Table **4**).

An additional analysis according to time period of BLS consumption (age 10 to 12, around the age 20, and 10 to 15 years prior to the study) showed adjusted ORs of BLS consumption ≥ four times per week to BLS consumption < four times per week were 0.86 (95% CI, 0.60–1.23; p = 0.41), 0.58 (0.37–0.92, p = 0.019) and 0.84 (0.57–1.24, p = 0.38), respectively.

## 4 DISCUSSION

Strengths of this study include (1) robustness of data, which were mostly comparable across the sensitivity analyses with different adjustments for confounders, (2) identification of known risk factors such as family history of breast cancer and history of benign tumor, (3) being a population-based study enrolling participants from multiple areas in Japan, (4) smaller biases due to an interview survey compared with a self-administered questionnaire survey, and (5) successful interviewer blinding. The interviewers were asked whether they had found out the case/control identity of the interviewees during interviews. They answered they had been able to identify cases and controls in 29% of the interview sessions, and they were incorrect about the case/control identification in 24% of the time. Therefore, the blinding was considered successful. As the limitations of this study recognized the following: While validated questionnaire forms [15] were used for the survey on current food consumption, the survey on past food consumption was not validated. However, the BLS distributor’s sales record and BLS consumption estimated based on the completed questionnaire forms were cross-checked and proven highly consistent [5]. The questionnaire response rate was low among controls (884/8166), possibly affecting the generalizability of the study conclusion. Controls were better educated on average and may have better understood the meaning of this study and have been willing to participate as controls. However, the educational background of participants was adjusted in the statistical model. According to Yakult Honsha data, purchase of BLS was not associated with household income, which is generally correlated with educational background.

Daily consumption of BLS since adolescence had a significant inverse association with early breast cancer occurrence. A significant inverse association was also seen between consumption of soy isoflavones and breast cancer occurrence. The results are consistent with those from a case-control study conducted by Hirose and colleagues [11] in which the OR for premenopausal breast cancer in the highest tertile of soy isoflavone consumption against the lowest tertile was estimated at 0.44 (95% CI, 0.22–0.89). BLS consumption increases the NK cell activity and boosts the immune system in human [9]. A chemical carcinogenesis study in mice showed oral intake of *L. casei* Shirota inhibited carcinogenesis by enhancing the NK cell activity [19]. Increased NK cell activity and isoflavone metabolisms [20] are both potential underlying mechanisms of the breast cancer preventive effect of BLS. Soy isoflavones and their metabolites have been shown to prevent breast cancer, prostate cancer and osteoporosis in a number of epidemiological studies. Having a higher affinity for estrogen receptor β and a stronger antioxidative activity compared with other isoflavone derivatives, a daidzein metabolite equol plays an important role in cancer prevention [21, 22]. Recently, an increase in the population level of intestinal lactobacilli was shown to potentially activate the intestinal isoflavone metabolism [23, 24]. The analyses in this study suggested a biological interaction between BLS and soy isoflavones. Just as in women who consume more soy isoflavones, breast cancer may be prevented in women who consume BLS even if they consume little soy isoflavones. The interaction between the intestinal flora and the isoflavone metabolism may also be involved in the mechanism. Further studies are warranted.

So far no prospective study in human has evaluated how BLS consumption changes the intestinal flora and equol production. Now intestinal flora can be identified using an inexpensive new technology that produces results quickly, which is based on the reverse transcription-quantitative polymerase chain reaction analysis of microbacterial rRNA in human feces [25]. This technology may be useful in future studies.

The stronger inverse association shown in our main analysis is consistent with the results of epidemiological studies showing an association between soy consumption in adolescence and decrease in breast cancer risk [26, 27] as well as the results of a breast cancer/prostate cancer prevention study in animals [28]. A subgroup analysis showed an inverse association between daily BLS consumption and breast cancer occurrence irrespective of menopausal status. The inverse association was strong and statistically significant in postmenopausal women.

## 5 CONCLUSION

This population-based case-control study in Japanese women showed an inverse association between BLS consumption since adolescence and breast cancer occurrence. Soy isoflavone consumption was also inversely associated with breast cancer occurrence as shown in the previous studies. 

Despite the study design that did not allow to indicate the recommended amount of probiotic beverages and soy isoflavone for the prevention of breast cancer, our study results suggested the benefit of consuming these products. Further studies may be able to recommend the lifestyle modification with diet including consumption of BLS and soy isoflavone.

## Figures and Tables

**Table 1.** Baseline Characteristicss

	Case (n=306)		Control (n=662)	
Age (years)	48	(4·1)	47	(3·7)
40-49	199	(65·0%)	461	(69·6%)
50-55	107	(35·0%)	201	(30·4%)
Educational background				
Other than college/graduate	264	(86·3%)	518	(78·3%)
College/graduate school or above	42	(13·7%)	144	(21·8%)
Physical activity level (METs/day)	26	(13·3)	27	(12·8)
Benign mammary tumor	51	(16·7%)	41	(6·2%)
Family breast cancer history	29	(9·5%)	28	(4·2%)
Age at menarche (years)	13	(1·3)	13	(1·3)
Number of births	2	(1·0)	2	(1·1)
Breastfeeding experience	232	(75·8%)	528	(79·8%)
Menopause	111	(36·3%)	200	(30·2%)
Use of female sex hormone before menopause				
Not using	254	(83·0%)	553	(83·5%)
Currently using	52	(17·0%)	109	(16·5%)
Birth weight				
≥ 2500g	270	(88·2%)	584	(88·2%)
< 2500g	21	(6·9%)	48	(7·3%)
Unknown/data not available	15	(4·9%)	30	(4·5%)
BMI at the ave. age of 20(kg/m^2^)	20	(2·4)	20	(2·2)
Smoking	38	(12·4%)	78	(11·8%)
Energy intake (1000kcal/day)	2.16	(0·8)	2.14	(0·8)

Data are n (%) or mean (SD).

**Table 2.** Hormone Receptor Status in Cases

	Case (n=306)	
Estrogen receptor		
Negative	28	(9.2%)
Positive	259	(84.9%)
Unknown	19	(6.2%)
Progesterone receptor		
Negative	60	(19.6%)
Positive	227	(74.2%)
Unknown	19	(6.2%)
HER2 receptor		
Negative	222	(72.6%)
Positive	36	(11.8%)
Unknown	48	(15.7%)

Data are n (%).

**Table 3.** Crude and Adjusted Odds

	Case (n=306)	Control (n=662)	Crude Odds Ratio‡				Adjusted Odds Ratio*			
			OR	95%CI		p	OR	95%CI		p
Probiotic beverage										
<4 times	88.9%	83.8%	Reference.			0.061	Reference.			0.048
≥ 4 times	11.1%	16.2%	0.66	0.43	1.02		0.65	0.42	1.00	
Soy isoflavone										
Q1（<18.76mg/day)	33.0%	24.9%	Reference.			0.0012**	Reference.			0.0002**
Q2 (18.76-<28.81mg/day)	25.8%	25.1%	0.74	0.49	1.10		0.76	0.52	1.13	
Q3 (28.81-<43.75mg/day)	21.6%	24.9%	0.58	0.38	0.88		0.53	0.35	0.81	
Q4 (43.75mg/day-)	19.6%	25.1%	0.52	0.34	0.79		0.48	0.31	0.73	

‡ Calculated using conditional logistic regression. 304 cases and 630 controls were matched for age and residential area. 2 cases and 32 controls were unmatched and excluded from the analysis.

* Calculated using conditional logistic regression. 306 cases and 661 controls were matched for residential area and adjusted for age (40s and 50s), educational background, physical activity level, benign mammary tumor, family breast cancer history, age at menarche, number of births, breastfeeding experience, use of female sex hormone before menopause, birth weight, Body Mass Index around the age of 20, smoking and energy intake. One control was excluded due to lack of adjustment factor.

** Trend P calculated from the linear score. (0=Q1, 1=Q2, 2=Q3, 3=Q4)

**Table 4.** Subgroup Analyses of Premenopausal Women and Postmenopausal Women

	Premenopausal Women						Postmenopausal Women						
	Case	Control	Adjusted Odds Ratio*				Case	Control	Adjusted Odds Ratio*				
	(n=195)	(n=462)	OR	95% CI		p	(n=111)	(n=200)	OR	95% CI			p
Probiotic beverage													
<4 times	88.2%	83.1%	Reference.			0.35	90.1%	85.5%	Reference.				0.046
≥ 4 times	11.8%	16.9%	0.78	0.46	1.32		9.9%	14.5%	0.43		0.19	0.99	

* Calculated using conditional logistic regression. Each subgroup was matched for residential area and adjusted for age (40s and 50s), educational background, physical activity level, benign mammary tumor, family breast cancer history, menarche, number of births, breastfeeding, use of female hormone, birth weight, Body Mass Index around the age of 20, smoking and energy intake. One control was excluded due to lack of adjustment factor in the analysis of premenopausal women.
